# Supplementary material for: A meta-analysis of birth-origin effects on reproduction in diverse captive environments
Source: Nat Commun. 2018 Mar 13;9:1055. doi: 10.1038/s41467-018-03500-9 (PMC5849764; doi:10.1038/s41467-018-03500-9)
Supplement: Supplementary file 1 — Supplementary Information [file 41467_2018_3500_MOESM1_ESM.pdf]

Supplementary Note 1

In testing for possible sources of publication bias in our dataset, we observed that the relationship between year of publication and effect size was not statistically significant (posterior mode slope estimate = -0.015, 95% HPD CI: [-0.05, 0.03], Supplementary Fig. 1a). This suggests no evidence of time-lag bias, such as may occur if studies with larger, significant effect sizes are published and dominate the literature before insignificant results, which may take longer to publish, appear[1]. Egger's regression testing for the symmetry of the funnel plot of the meta-analytic residuals from the overall model against their precision indicated statistically significant asymmetry (t_{115} = -0.825, P = 0.0077, Supplementary Fig. 1b). While funnel plot asymmetry is used to identify publication bias, it can also be the result of true heterogeneity or chance[1]. Our dataset was characterized by high total heterogeneity (I^2_{total} = 94%), and high heterogeneity between studies (I^2_{study} = 70%), so it is plausible that the funnel plot asymmetry is not reflective of publication bias. Any difference in reproductive success between wild-born and captive-born animals is likely to be of interest to captive managers. Trim-and-fill analysis estimated two effect sizes missing from the right hand side of the right side of the distribution, however this was not statistically significant (P = 0.125), and the estimated adjustment was small (lnOR = 0.038) and does not qualitatively influence our results.

We observed an outlier in our dataset[2], with an effect size of lnOR = -9.8 (Supplementary Fig. 1c). As such, we re-ran all of the above models excluding this data point. All model results were qualitatively similar, and the Egger's regression still identified funnel plot asymmetry (t_{114} = -1.06, P = 0.002). However, the trim-and-fill analysis estimated no missing effect sizes. Overall therefore, it is unlikely that publication bias is driving our main results.

Supplementary Note 2

We observed a strong linear correlation between the absolute mean of an estimate and its standard deviation on the natural log scale, as expected under Taylor's Law[3] (Supplementary Fig. 4). To recover missing standard deviations for 17 comparisons, we performed 20 imputations of missing log standard deviations using the 'mice' package in R[4] and exponentiated the resulting values to calculate effect sizes. These additional 17 comparisons were added to the 115 comparisons in the main dataset, resulting in a total of 132 comparisons, and all meta-analyses re-run. We pooled the posterior estimates from each of the 20 imputations to obtain the posterior mode, and 95% HPD CIs were calculated on the pooled data using the 'hdi' function in the 'HDInterval' package[5]. An additional four species and four papers that were not included in the main analysis were included by imputation, resulting in 48 species and 43 papers in total. The four additional species (cynomolgus macaque *Macaca fascicularis*, cheetah *Acinonyx jubatus*, American lobster *Homarus americanus*, and oval squid *Sepioteuthis lessoniana*) included using multiple imputation were distributed across the taxonomic tree represented by the main analysis. The imputed comparisons covered aquaculture, conservation and research environments, and four of the five reproductive trait categories (all except offspring survival) (Supplementary Table 4).

All estimated effects were of similar magnitude to the main analysis for all models (Supplementary Table 4). Statistical significance of the overall result, the effects from the model fitted with the 'captive environment' moderator, and the offspring quality and offspring survival traits remained the same as our main analysis. The estimated effects were in the same direction as the original analysis, with the exception of reproductive phenology, which became positive but remained close to zero and not statistically significant (lnOR = 0.14 [imputed] vs. -0.04 [main analysis]). However, the effects based on imputation were estimated with poorer precision than in the main analysis, as evident from the widened 95% HPD CIs for each result (Supplementary Table 4).

We also considered whether it was possible to impute missing sample sizes, although the relationship between means and samples sizes was less clear than the relationship between mean and standard deviation (Supplementary Fig. 4). Imputing sample size provided a further 24 comparisons to the 132 noted above (total N = 156). The posterior mode estimates were again similar and in the same direction as the original analysis, and uncertainty in the estimates did not improve with the inclusion of these additional values (data not shown). Nevertheless, because the estimated effects were similar across our datasets, we do not believe that our overall conclusions are biased by missing data.

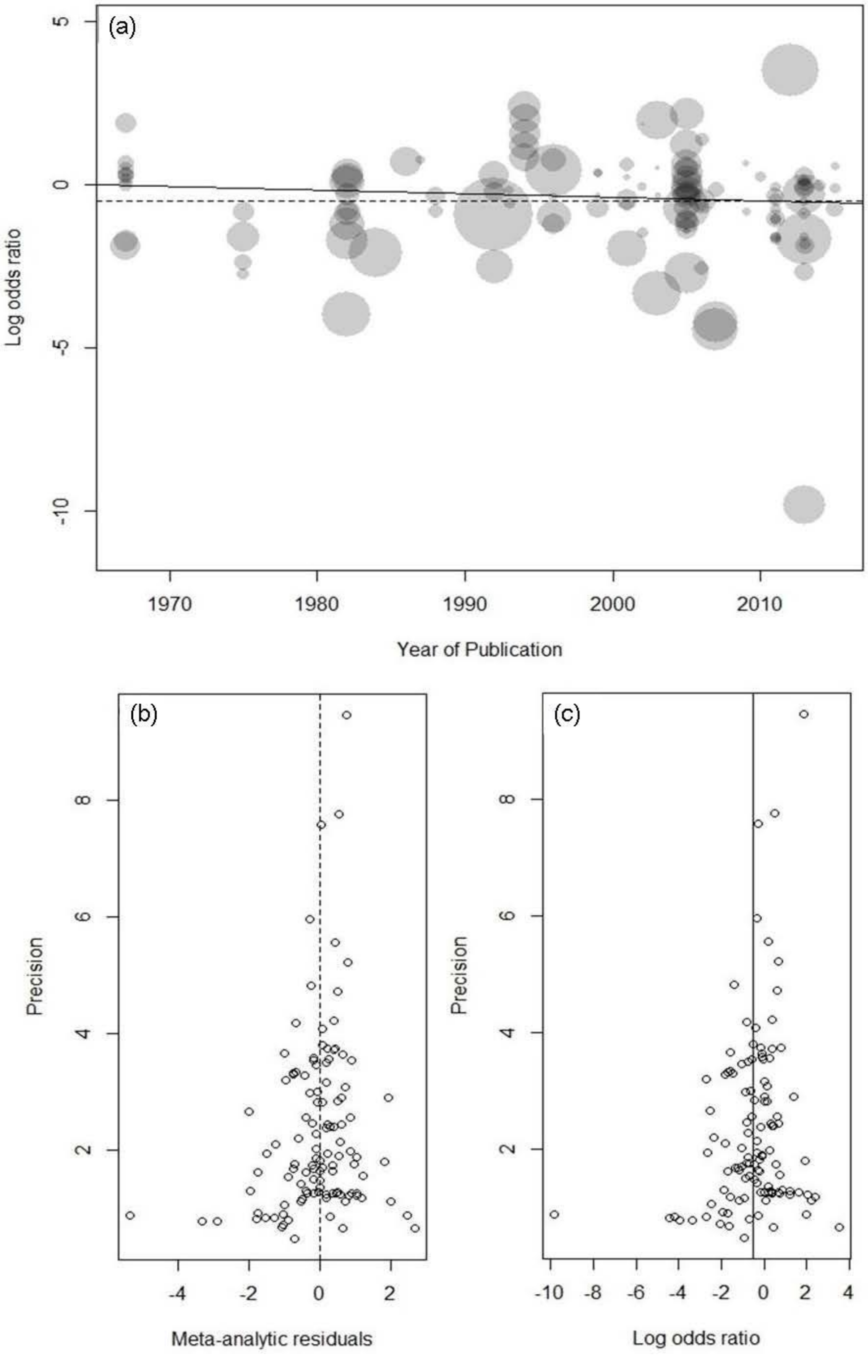

Supplementary Fig. 1. Tests for publication bias.
(a) Relationship between effect size (log odds ratio) and year of publication (indicated by solid line), to examine evidence of time-lag bias. Dashed line shows meta-analytic mean from the overall non-phylogenetic model. Point size is proportional to the variance of the effect size. (b) Funnel plot of meta-analytic residuals extracted from the overall non-phylogenetic model plotted against their precision $(1/\text{variance})^{1/2}$, with the dashed line at 0. (c) Funnel plot of effect sizes (log odds ratios) plotted against their precision, with solid line showing meta-analytic mean.

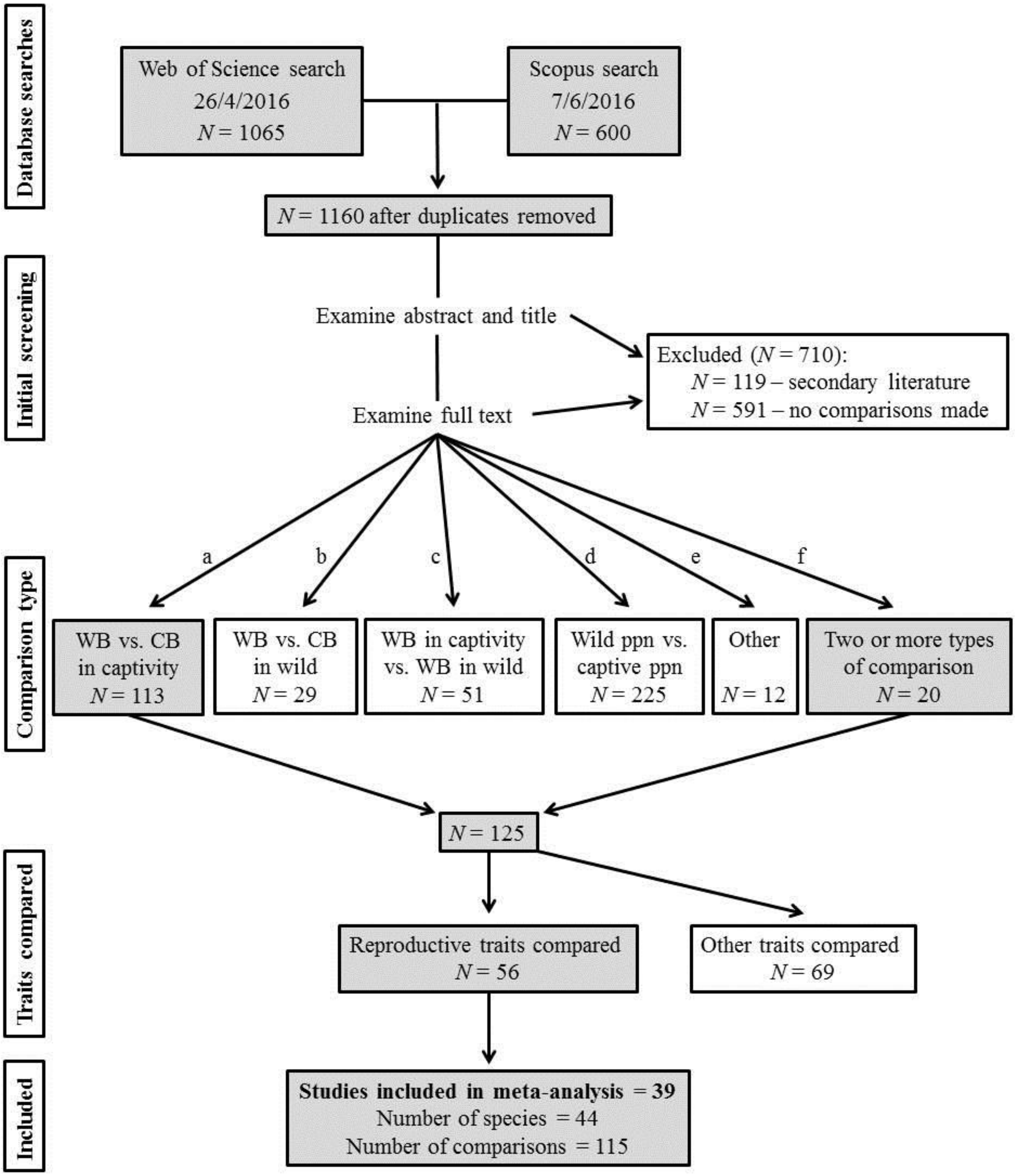

Supplementary Fig. 2. PRISMA flowchart[6] of the overall literature filtering strategy. *N* refers to the number of papers included at each stage of filtering. Shaded boxes represent the papers under consideration for inclusion in the systematic review and meta-analysis at each stage of filtering. a – f are the categories of comparison type, referred to in Methods. (WB = wild-born, CB = captive-born, ppn = population).

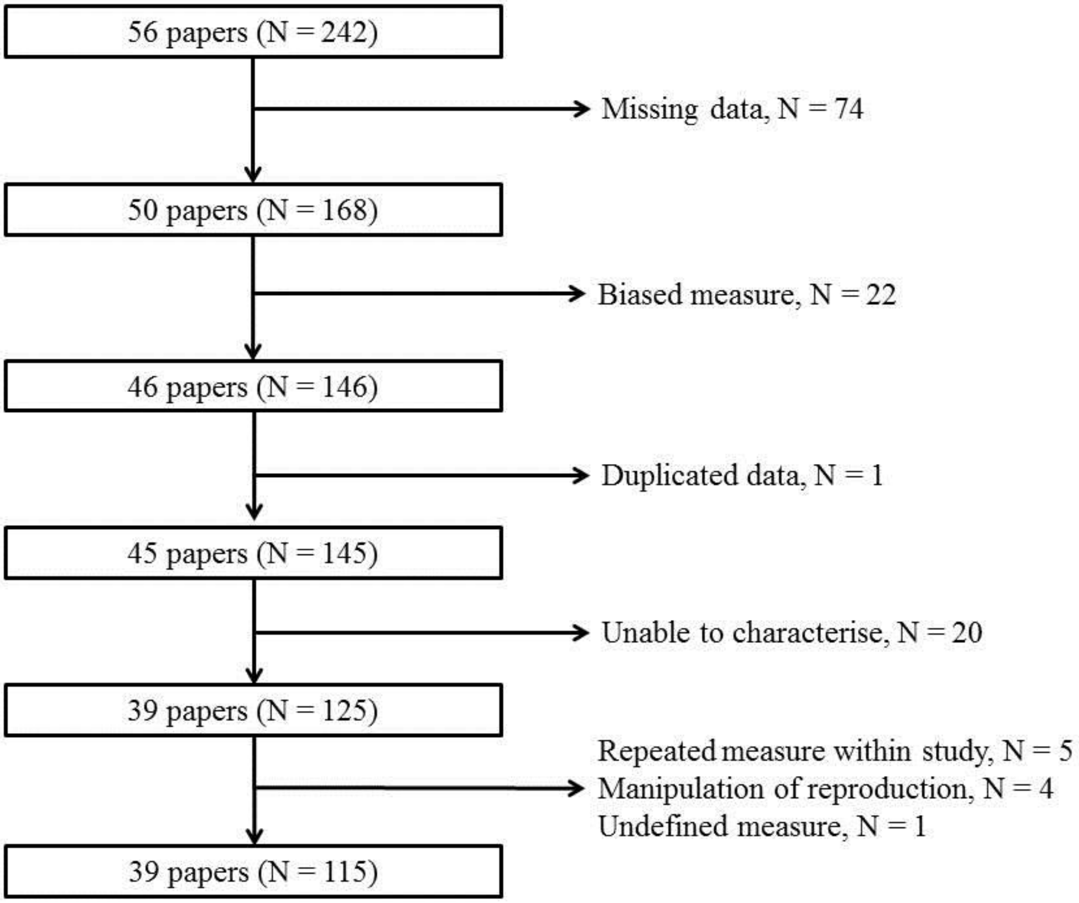

Supplementary Fig. 3. Filtering strategy with reasons for excluding comparisons and therefore papers from the analysis; *N* = number of comparisons.

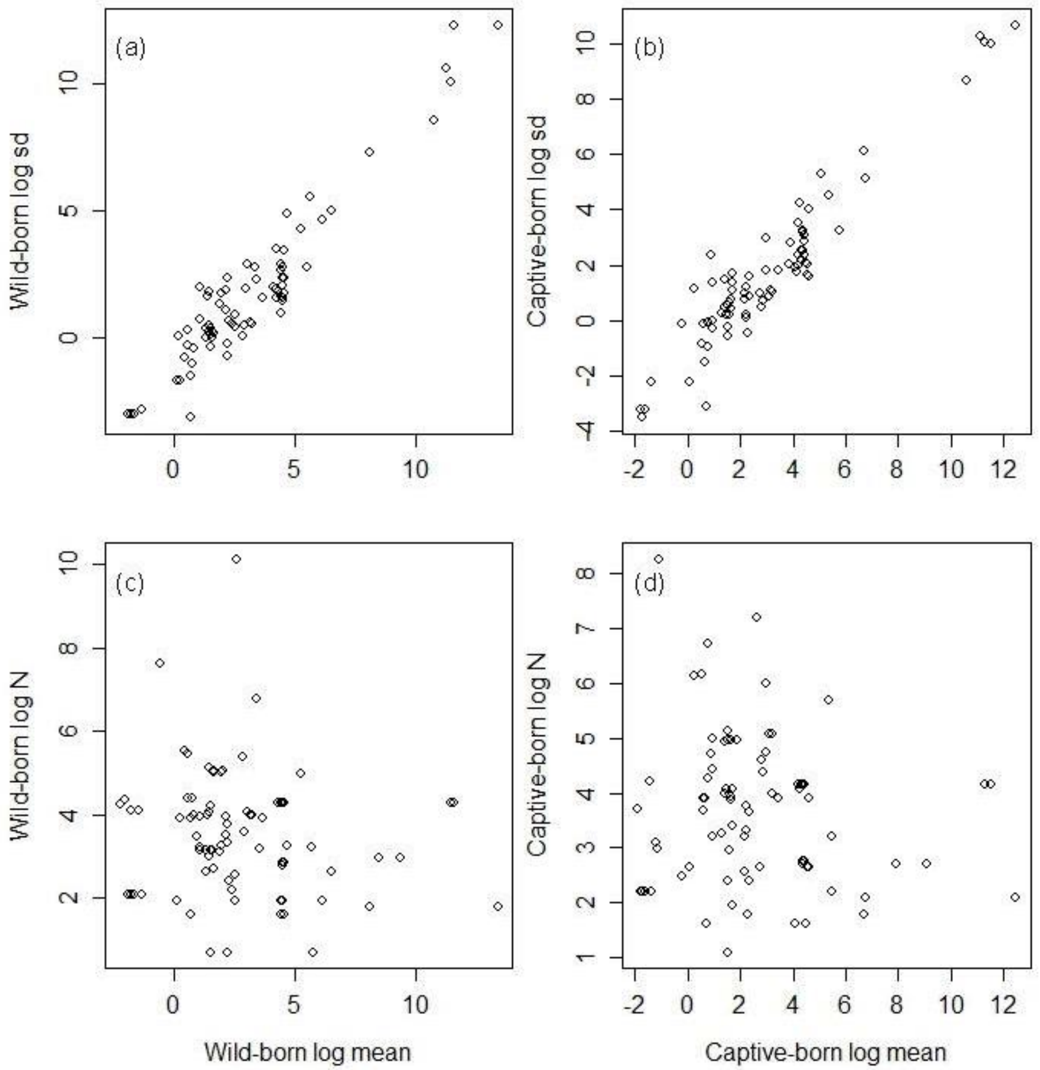

Supplementary Fig. 4. Relationships between the mean and standard deviation (*sd*) or sample size (*N*) of wild-born and captive-born continuous comparisons (*N* = 67) on the natural log scale. Relationships between the log standard deviation and log mean of (a) wild-born and (b) captive-born continuous comparisons. Relationships between log sample size and log mean of (c) wild-born and (d) captive-born continuous comparisons.

Supplementary Table 1. Extended heterogeneity (I^2) statistics for the overall non-phylogenetic model and the overall model + phylogeny.

	Heterogeneity (%)			
	Total	Phylogeny	Study ID	Residual variance
Overall model	93.736	-	70.185	19.332
Overall model + phylogeny	94.339	0.290	67.307	24.235

Supplementary Table 2. Assignment of captive-born to wild-born comparisons (as defined by original authors of publications) to reproductive trait type categories used in the meta-analyses, and direction of each effect on overall reproductive success.

Trait type	Comparisons	Direction of effect	*N*
Fertility/ hatchability	Fertility (of the egg, clutch, or spawn; across years; live born offspring/female/reproductive year)	+	8
	Proportion of successful hatching (out of total incubated eggs, fertile eggs, clutch, or spawning; across years)	+	6
	Reproductive success (i.e. binary statistic indicating producing at least one offspring for males, females, or pairs, or of these within a given time frame from pairing e.g. 6 months)	+	13
	Proportion of population with reproductive abnormalities (e.g. pathological lesions of reproductive tract)	-	1
	% normal sperm (visually) or % reactive sperm (undergo changes when in contact with egg)	+	2
Reproductive yield	Number of litters per pair	+	1
	Number of offspring (e.g. per female; or per individual in given time frame)	+	12
	Clutch/litter size (of 1st/2nd/3rd/4th litter; or litter size at weaning)	+	12
	Number of offspring surviving to a given time point (e.g. 5 years) per female per year	+	2
	Number of offspring produced per gram of body weight of female	+	1
Offspring quality	Proportion of offspring birth abnormalities (e.g. chondrodystrophy)	-	1
	Egg morphometric traits (e.g. mass, volume)	+	2
	Offspring size (hatch weight or length; body weight at weaning)	+	5
Offspring survival	Mortality rate (of embryos, neonates, or infants; also described as prenatal, perinatal or postnatal mortality)	-	8
	Juvenile mortality rate at a given time point (e.g. 1 week, 2 weeks, 6 months)	-	11
	Juvenile survival (to a given time point or developmental stage)	+	5
	Incidence of cannibalism/abandonment of young by parent	-	3
	Proportion of young successfully reared (live offspring out of total)	+	3
	Stillbirth/abortion rate	-	3
Reproductive phenology	Breeding interval (between pairing and first litter; interbirth or spawning interval)	-	7
	Rate of production of offspring (spawning rate)	+	3
	Mating rate (e.g. matings per female per month)	+	2
	Age at first parturition	-	4

Positive effect (+) results in increased overall reproductive success, negative effect (-) results in decreased reproductive success. *N* is number of comparisons within each trait type category (total *N* = 115).

Supplementary Table 3. Generation (F) of the captive-born population compared to the wild-born population specified within the study grouped for each study environment category.

	Aquaculture	Conservation	Research	Other	Total
No generation specified	7	47	25	1	80
F1	12	0	5	0	17
F1-F2	1	0	8	0	9
F1-F3	3	2	1	0	6
F1-F4	0	2	1	0	3
Total	23	51	40	1	115

Data are the number of comparisons/effect sizes within each group.

Supplementary Table 4. Meta-analytic effect size estimates of differences in reproductive success between wild-born and captive-born animals in captive environments for the dataset including the original comparisons (*N* = 115) and the additional imputed comparisons (*N* = 17).

	Posterior mode (lnOR)	Lower 95% HPD CI	Upper 95% HPD CI	*N*
Overall model*	-0.67	-1.83	-0.04	132
Overall model + phylogeny	-0.96	-2.85	0.42	132
Captive environment				
*Aquaculture**	-1.70	-3.78	-0.18	25
Conservation	-0.11	-1.38	1.24	59
Research	-0.78	-2.97	0.17	47
Other	2.00	-3.50	7.04	1
Trait type				
Fertility & hatchability	-0.92	-2.23	0.03	31
Reproductive yield	-0.84	-1.95	0.13	38
*Offspring quality**	-1.59	-3.27	-0.32	8
*Offspring survival**	-1.21	-2.49	-0.29	33
Reproductive phenology	0.14	-1.10	1.04	22

Posterior mode gives the meta-analytic log odds ratio (lnOR) estimate from the MCMCglmm models, with lower and upper 95% higher posterior density credible intervals given. Estimates with the 95% HPD CI excluding zero are marked with *.

Supplementary Table 5. Publications comparing reproductive traits in wild-born and captive-born animals in captive environments excluded from main analysis and reasons for their exclusion.

Publication	Species	Reason(s) for exclusion
Clubb *et al.* [7]	African elephant, *Loxodonta africana*, Asian elephant, *Elephas maximus*	Missing data (analysis of juvenile mortality not conducive to calculation of effect sizes)
Curry *et al.* [8]	Polar bear, *Ursus maritimus*	Comparison biased by opportunity to breed (e.g. total lifetime number of litters produced)<br>Direction of effect on productivity can't be characterized for day of parturition or offspring sex ratio<br>Missing data (no raw data or only P-values reported for litter size, incidence of stillbirths, neonatal mortality, juvenile survival and inter-birth interval)
Gupta [9]	Round Island gecko, *Phelsuma guentheri*	Direction of effect cannot be characterized for age-specific fecundity
Ikeda *et al.* [10]	Oval squid, *Sepioteuthis lessoniana*	Missing data (no error or sample size reported for age at first spawning or number of egg cases/female)
Keeley *et al.* [11]	Tasmanian devil, *Sarcophilus harrisii*	Data are encompassed in a more recent and larger sample size study [Hogg et al. (2015)]
Kirkland and Linzey [12]	Deer mouse, *Peromyscus maniculatus*	Missing data (no error or sample size reported for litter size)
Levallois & de Marigny [13]	Cynomolgus macaque, *Macaca fascicularis*	Missing data (no error reported for inter-birth interval, no sample size reported for neonatal mortality, stillbirth incidence or proportional birth rate)
Mace [14]	Western lowland gorilla, *Gorilla gorilla*	Comparison biased by opportunity to breed (e.g. total offspring produced per male/female)
Mar [15]	Asian elephant, *Elephas maximus*	Direction of effect cannot be characterized for age-specific fecundity or offspring sex ratio<br>Missing data (analysis of interbirth interval not conducive to effect size calculation)

Marker-Kraus [16]	Cheetah, *Acinonyx jubatus*	Missing data (no error or sample size for age at first or last parturition for males and females)<br>Comparison biased by opportunity to breed (e.g. total number of litters and total number of offspring produced in a lifetime)
Meng *et al.* [17]	Alpine musk deer, *Moschus sifanicus*	Direction of effect cannot be characterized for mating date
Mooney & Lee [18]	Woolly monkey, *Lagothrix lagotricha*	Missing data (only *P*-value reported for infant mortality, analysis of age at first birth and interbirth interval not conducive to effect size calculation)<br>Biased measures of reproductive success (e.g. number of population having more than one reproductive event is biased by opportunity to breed)
Rasweiler & Badwaik [19]	Short-tailed fruit bat, *Carollia perspicillata*	Direction of effect cannot be characterized for gestation length
Stuermer *et al.* [20]	Mongolian gerbil, *Meriones unguiculatus*	Missing data (no error or sample size reported for litter size)
Talbot *et al.* [21]	American lobster, *Homarus americanus*	Comparison biased by opportunity to breed (e.g. total egg production and total number of eggs attached)<br>Missing data (no error reported for number of eggs extruded/female or number of eggs attached/female)
Vermeer & Devreese [22]	Western lowland gorilla, *Gorilla gorilla*	Missing data (only *P*-value reported for infant mortality)<br>Direction of effect cannot be characterized for offspring sex ratio
Yu [23]	Golden monkey, *Rhinopithecus roxellanae*	Missing data (no sample size reported for reproductive rate)

See Methods for details of inclusion/exclusion criteria. Note that comparing captive-born and wild-born animals may not have been the primary aim of some studies with missing data.

Supplementary References

1. Higgins, J. P. T., Green S. *Cochrane handbook for systematic reviews of interventions*. The Cochrane Collaboration www.handbook.cochrane.org (2011).

2. Murugan, A., Dhanya S., Pawar H., Sreepada R. A., Rajagopal S., Balasubramanian T. Preliminary observation on breeding three spotted seahorse, *Hippocampus trimaculatus* (Leach, 1814), solely fed with wild caught amphipods under ex - situ condition. *Indian J. Anim. Sci.* **83**, 204-208 (2013).

3. Taylor, L. R. Aggregation, variance and the mean. *Nature* **189**, 732-735 (1961).

4. van Buuren, S., Groothuis-Oudshoorn K. mice: Multivariate imputation by chained equations in R. *J. Stat. Softw.* **45**, 1-67 (2011).

5. Meredith, M., Kruschke J. HDInterval: Highest (posterior) density intervals. https://CRAN.R-project.org/package=HDInterval (2016).

6. Moher, D., Liberati A., Tetzlaff J., Altman D. G. Preferred reporting items for systematic reviews and meta-analyses: The PRISMA Statement. *PLOS Med.* **6**, e1000097 (2009).

7. Clubb, R., Rowcliffe M., Lee P., Mar K. U., Moss C., Mason G. J. Compromised survivorship in zoo elephants. *Science* **322**, 1649-1649 (2008).

8. Curry, E., Safayi S., Meyerson R., Roth T. L. Reproductive trends of captive polar bears in North American zoos: a historical analysis. *J. Zoo Aquarium Res.* **3**, 99-106 (2015).

9. Gupta, B. K. A comparison between fecundity rates in captive hatched and wild collected endangered Round Island day geckos (*Phelsuma guentheri*) at the Jersey Wildlife Preservation Trust, U.K. *Anim. Keepers' Forum* **21**, 283-286 (1994).

10. Ikeda, Y., Ueta Y., Anderson F. E., Matsumoto G. Reproduction and life span of the oval squid *Sepioteuthis lessoniana* (Cephalopoda: Loliginidae): comparison between laboratory-cultured and wild-caught squid. *Mar. Biodivers. Rec.* **2**, e50 (2009).

11. Keeley, T., O'Brien J. K., Fanson B. G., Masters K., McGreevy P. D. The reproductive cycle of the Tasmanian devil (*Sarcophilus harrisii*) and factors associated with reproductive success in captivity. *Gen. Comp. Endocrinol.* **176**, 182-191 (2012).

12. Kirkland, G. L., Jr., Linzey A. V. Observations on the breeding success of the deer mouse, *Peromyscus maniculatus nubiterrae*. *J. Mammal.* **54**, 254-255 (1973).

13. Levallois, L., de Marigny S. D. Reproductive success of wild-caught and captive-bred cynomolgus macaques at a breeding facility. *Lab Animal* **44**, 387-393 (2015).

14. Mace, G. M. The genetic and demographic status of the western lowland gorilla (*Gorilla gorilla gorilla*) in captivity. *J. Zool.* **216**, 629-654 (1988).

15. Mar, K. U. Birth sex ratio and determinants of fecundity in female timber elephants of Myanmar. *Gajah* **38**, 8-18 (2013).

16. Marker-Kraus, L. History of the cheetah *Acinonyx jubatus* in zoos 1829-1994. *Int. Zoo Yearb.* **35**, 27-43 (1997).

17. Meng, X. X., Yang Q. S., Xia L., Feng Z. J., Jiang Y. W., Wang P. M. The temporal estrous patterns of female alpine musk deer in captivity. *Appl. Anim. Behav. Sci.* **82**, 75-85 (2003).

18. Mooney, J. C., Lee P. C. Reproductive parameters in captive woolly monkeys (*Lagothrix lagotricha*). *Zoo Biol.* **18**, 421-427 (1999).

19. Rasweiler, J. J., Badwaik N. K. Delayed development in the short-tailed fruit bat, *Carollia perspicillata*. *J. Reprod. Fertil.* **109**, 7-20 (1997).

20. Stuermer, I. W., Plotz, K., Leybold, A., Zinke, O., Kalberlah, O., Samjaa, P., Scheich, H. Intraspecific allometric comparison of laboratory gerbils with Mongolian gerbils trapped in the wild indicates domestication in *Meriones unguiculatus* (Milne-Edwards, 1867) (Rodentia: Gerbillinae). *Zool. Anz.* **242**, 249-266 (2003).

21. Talbot, P., Thaler C., Wilson P. Spawning, egg attachment and egg retention in captive lobsters (*Homarus americanus*). *Aquaculture* **37**, 239-249 (1984).

22. Vermeer, J., Devreese L. Birth sex ratio, infant mortality and rearing type in captive western lowland gorillas. *J. Zoo Aquarium Res.* **3**, 6-10 (2015).

23. Yu, Z.-Y. Analysis of demography and genetics of captive population of golden monkey. *Chin. J. Zool.* **39**, 45-49 (2004).
