# Supplementary material for: A meta-analysis of birth-origin effects on reproduction in diverse captive environments
Source: Nat Commun. 2018 Mar 13;9:1055. doi: 10.1038/s41467-018-03500-9 (PMC5849764; doi:10.1038/s41467-018-03500-9)
Supplement: Supplementary file 3 — Description of Additional Supplementary Files [file 41467_2018_3500_MOESM3_ESM.pdf]

Description of Additional Supplementary Files

File Name: **Supplementary Data 1**
Description: Excel file containing the data extracted and used for the meta-analysis. The first sheet contains the main dataset: data extracted from the 39 studies (115 comparisons). The second sheet contains data extracted from the additional 41 comparisons with missing data that could be recovered by multiple imputation. A key to the column headings can be found on the third worksheet of the Supplementary Data 1 file.

File Name: **Supplementary Data 2**
Description: Annotated R code used for conducting analyses.
